# The various therapeutic applications of the medical isotope holmium-166: a narrative review

**DOI:** 10.1186/s41181-019-0066-3

**Published:** 2019-08-05

**Authors:** Nienke J. M. Klaassen, Mark J. Arntz, Alexandra Gil Arranja, Joey Roosen, J. Frank W. Nijsen

**Affiliations:** 1 Department of Radiology and Nuclear Medicine, Radboud University Medical Center, Radboud Institute for Health Sciences, Geert Grooteplein Zuid 10, 6525 GA Nijmegen, The Netherlands; 2 Department of Pharmaceutics, Utrecht Institute for Pharmaceutical Sciences (UIPS), Science for Life, Faculty of Science, Utrecht University, 3508 TB Utrecht, The Netherlands; 3 Department of Radiation Science and Technology, Delft University of Technology, Mekelweg 15, 2629 JB Delft, The Netherlands

**Keywords:** Holmium-166, Holmium, Lanthanide, Radiation therapy, SIRT, Microspheres, Chitosan, DOTMP

## Abstract

Over the years, a broad spectrum of applications of the radionuclide holmium-166 as a medical isotope has been established. The isotope holmium-166 is attractive as it emits high-energy beta radiation which can be used for a therapeutic effect and gamma radiation which can be used for nuclear imaging purposes. Furthermore, holmium-165 can be visualized by MRI because of its paramagnetic properties and by CT because of its high density. Since holmium-165 has a natural abundance of 100%, the only by-product is metastable holmium-166 and no costly chemical purification steps are necessary for production of nuclear reactor derived holmium-166. Several compounds labelled with holmium-166 are now used in patients, such Ho^166^-labelled microspheres for liver malignancies, Ho^166^-labelled chitosan for hepatocellular carcinoma (HCC) and [^166^Ho]Ho DOTMP for bone metastases. The outcomes in patients are very promising, making this isotope more and more interesting for applications in interventional oncology. Both drugs as well as medical devices labelled with radioactive holmium are used for internal radiotherapy. One of the treatment possibilities is direct intratumoural treatment, in which the radioactive compound is injected with a needle directly into the tumour. Numerous other applications have been developed, like patches for treatment of skin cancer and holmium labelled antibodies and peptides. The second major application that is currently clinically applied is selective internal radiation therapy (SIRT, also called radioembolization), a novel treatment option for liver malignancies. This review discusses medical drugs and medical devices based on the therapeutic radionuclide holmium-166.

## Introduction

**Fig. 1:**
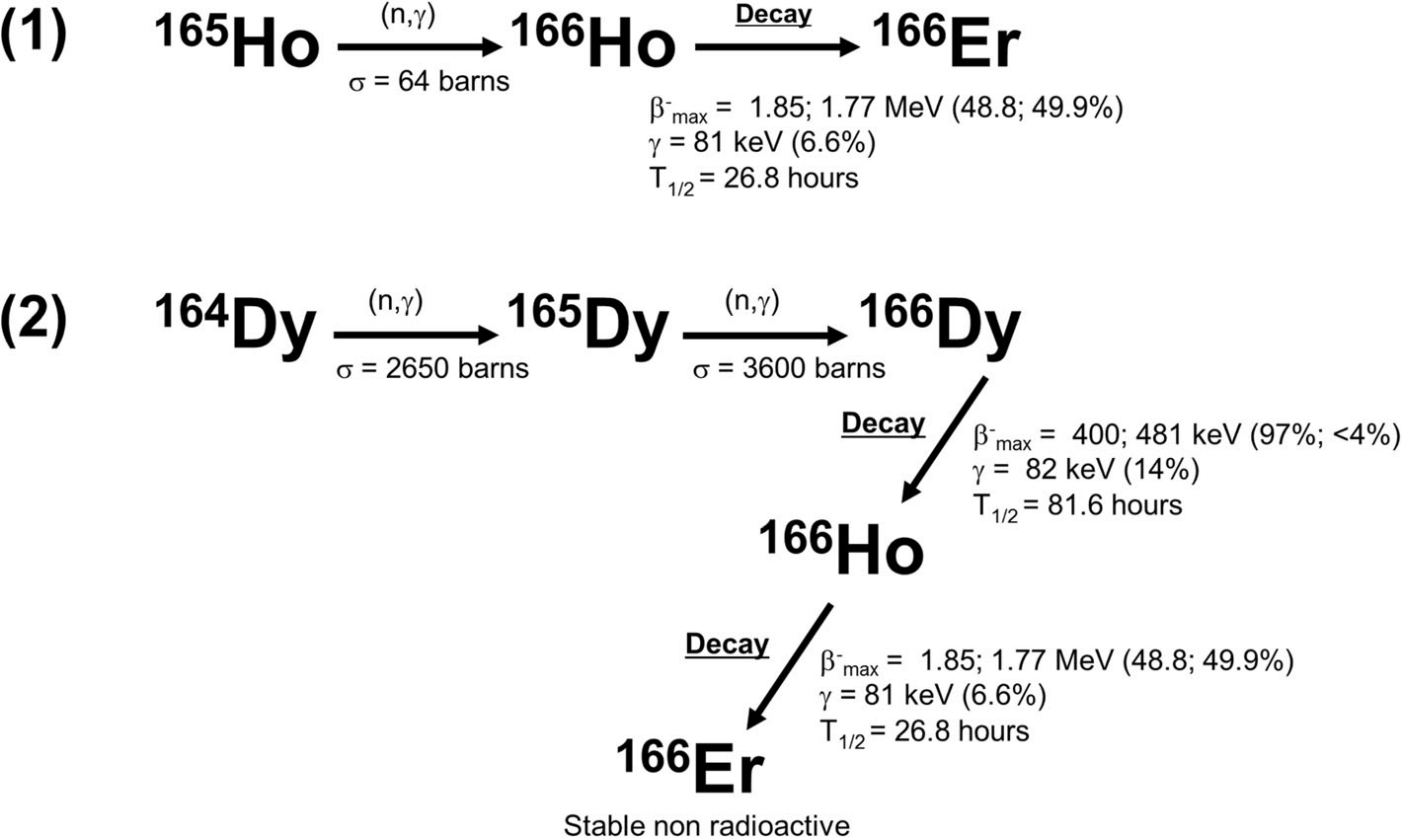
Diagrams of the production methods of (1) ^166^Ho and (2) ^166^Dy. Reactor neutron activated ^165^Ho will result in ^166^Ho with a high purity (1). The second method is via neutron activation of ^164^Dy by two neutrons. Dysprosium-164 has a natural abundance of 28.2% and enriched material will have a purity of over 90%. By capture of two neutrons, ^164^Dy will be converted into ^166^Dy which will decay into carrier-free ^166^Ho as the daughter radionuclide (^166^Dy/^166^Ho generator) (data were collected from the International Atomic Energy Agency Database: https://www-nds.iaea.org/)

Holmium is one of the 15 rare earth elements called lanthanides, a group of elements that has become an established source of radionuclides for nuclear diagnostic and therapeutic applications (Nayak and Lahiri 1999). Holmium-166 (^166^Ho) can be produced by two methods; neutron activation by (n, γ) irradiation in a nuclear reactor (Nayak and Lahiri 1999; Nijsen et al. 1999) or by neutron activation of dysprosium-164 (^164^Dy) (Nijsen et al. 2007) (Fig. 1). Because holmium-165 (^165^Ho) has a natural abundance of 100% and a cross section of 64 b (Foote Jr et al. 1953), it can be neutron activated in a relatively short neutron activation time resulting in ^166^Ho with a high purity of the isotope (Nijsen et al. 1999). The only by-product is metastable holmium-166 (^166m^Ho), approximately a factor 7 × 10^6^ times less than ^166^Ho. ^166m^Ho has a half-life of 1200 years and emits beta radiation and a number of gamma rays between 80 and 1563 keV (Nijsen et al. 2007; Hino et al. 2000; Bernardes 2001). The cross section of the ^165^Ho(n, γ)^166^Ho reaction is 64 b (Foote Jr et al. 1953) and the cross section of the ^165^Ho(n, γ)^166m^Ho reaction is around 3.4 b for thermal neutrons (Nethaway and Missimer 1968).

The other production option is via neutron activation of ^164^Dy by two neutrons following a (2n, γ) reaction forming dysprosium-166 (^166^Dy). The cross section of ^164^Dy is extremely high (2650 b). A second neutron irradiation on this instable nuclide is necessary to result in ^166^Dy. The radionuclide ^166^Dy will decay with a half-life of 81.5 h into ^166^Ho, during which two beta particles up to 481 keV are emitted (Smith et al. 1995).

The radionuclide ^166^Ho emits high-energy beta particles (1774.32 keV; yield 48.8% and 1854.9 keV; yield 49.9%) and gamma rays (80.57 keV; yield 6.7% and 1379.40 keV; yield 0.9% (only emissions are given with yield higher than 0.5%) and has a half-life of 26.8 h (Nijsen et al. 2007) (Fig. 1). The high energetic beta particles are responsible for the therapeutic effect and the gamma ray of 80.57 keV can be used for nuclear imaging purposes. Furthermore, ^165^Ho can be visualized by MRI because of its paramagnetic properties (Nijsen et al. 2004; Seevinck et al. 2007; Smits et al. 2012) and by CT because of its high density (Seevinck et al. 2007; Bakker et al. 2018).

As the half-life of ^166^Ho is 26.8 h, over 90% of the radiation is deposited in less than 4 days. The maximum tissue range of the beta particles in soft tissue is 8.7 mm, the average range 2.2 mm and 90% of the total radiation dose will be delivered in the first 2.1 mm (Johnson and Yanch 1991). This results in an interesting relatively high dose-rate if the same cumulative dose is given compared to other often used radioisotopes for cancer treatment, such as phosphor-32 (^32^P), yttrium-90 (^90^Y), iodine-131 (^131^I), lutetium-177 (^177^Lu) and rhenium-186 (^186^Re), with half-lives between 2.7 and 14.3 days.

**Fig. 2:**
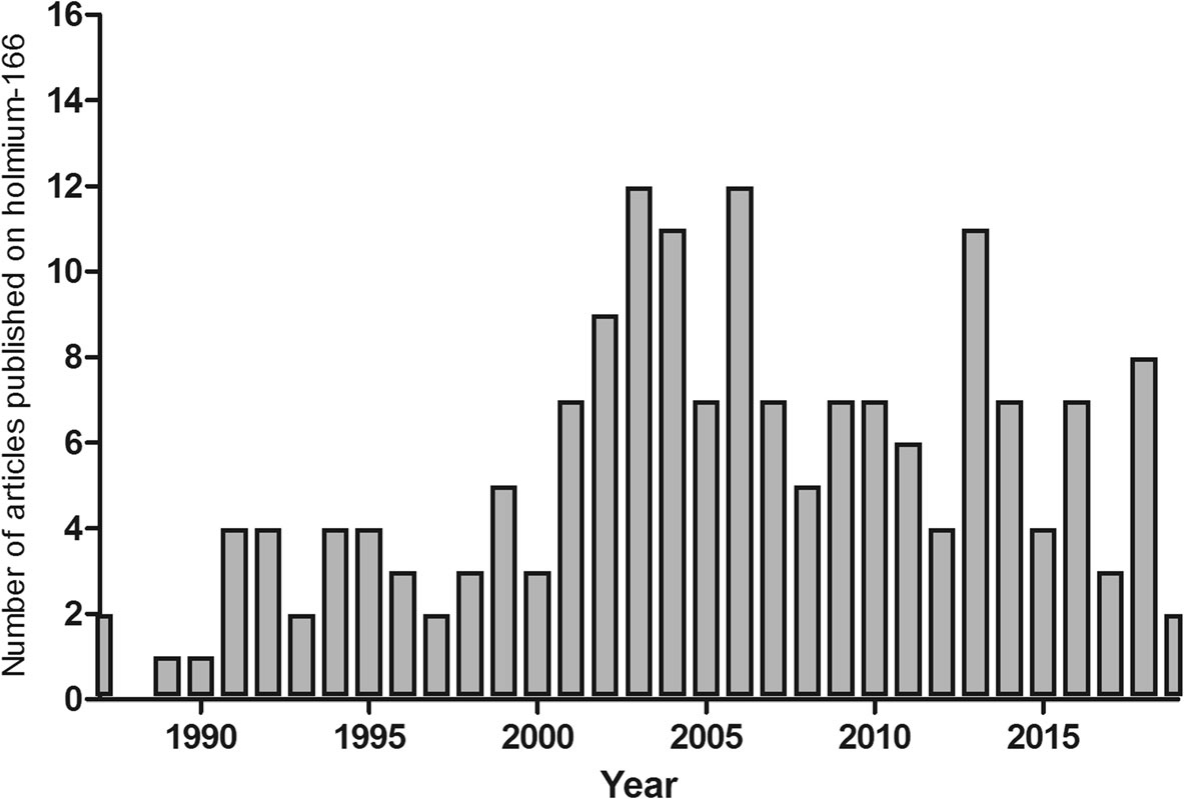
Number of ^166^Ho related publications over the years (search through PubMed)

**Fig. 3:**
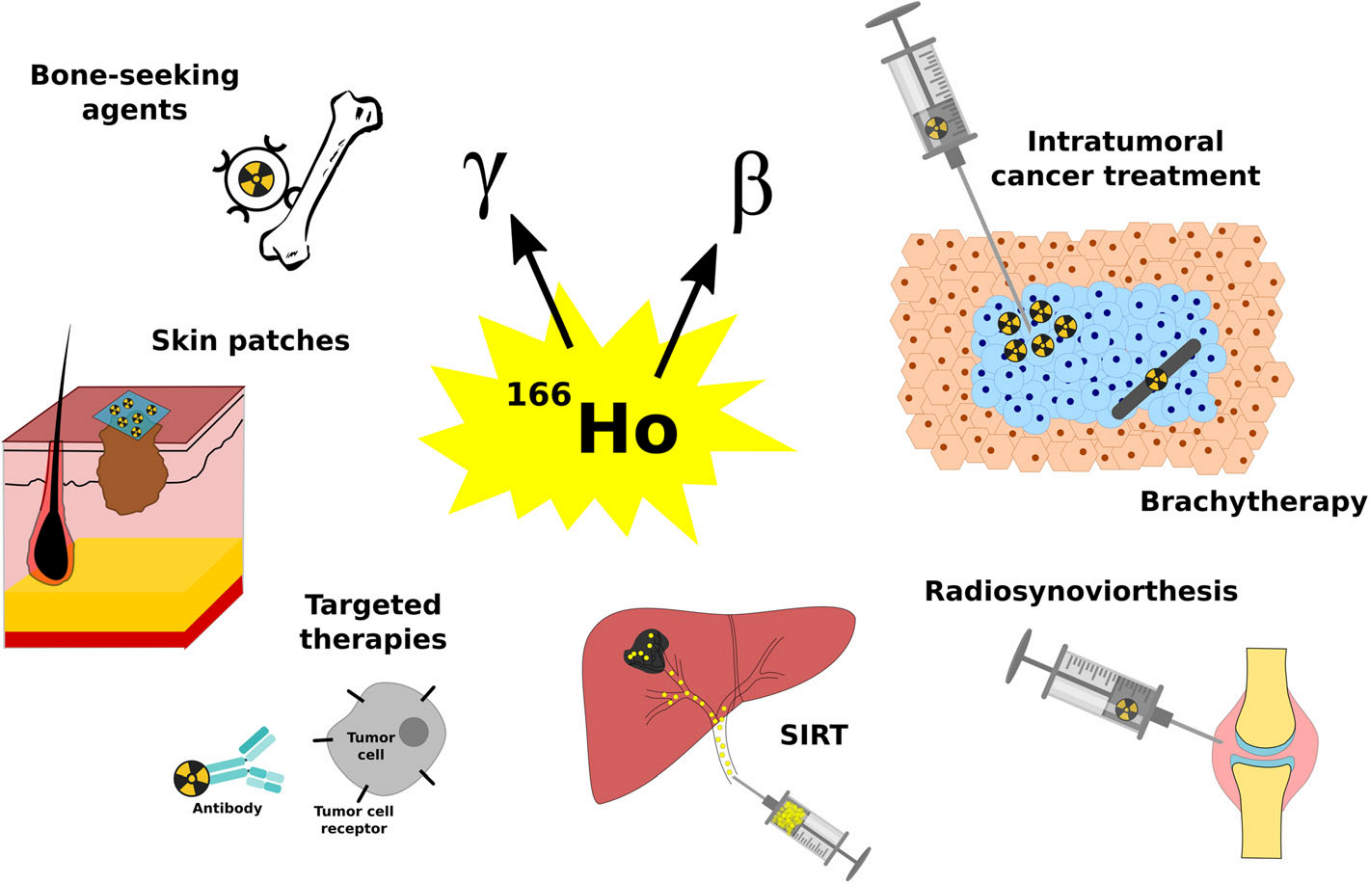
Schematic overview of medical applications of the isotope ^166^Ho

Over the last 30 years, 150 articles have been published on the use of ^166^Ho as a medical isotope. Especially since the start of this century interest in this radioisotope has been growing, resulting in around 6 publications per year (Fig. 2) and translated into the development of many systems loaded or conjugated to holmium (Table 1). Since the production of ^166^Ho has become more and more standardized, the number of clinical applications (Fig. 3) and clinical trials (Table 2) has been growing and several compounds are now used in patients: ^166^Ho-labelled microspheres for liver malignancies (Smits et al. 2012; Prince et al. 2018), ^166^Ho-labelled chitosan for hepatocellular carcinoma (HCC) (Sohn et al. 2009) and [^166^Ho]Ho DOTMP (1,4,7,10-tetraazacyclododecane-1,4,7,10-tetramethylene-phosphonic acid) for bone metastases (Denis-Bacelar et al. 2018). Thus, it can be expected that the significance of the use of this radioisotope will continue to grow rapidly. In this review, the many therapeutic applications of ^166^Ho that have been developed over the years are discussed.

**Table 1:** Representative examples of carriers that have been loaded or conjugated to holmium and to the dysprosium/holmium generator

Bone seeking agents	DOTMP	(Bayouth et al. 1995a; Bayouth et al. 1995b; Parks et al. 1993; Giralt et al. 2003; Breitz et al. 2006; Rajendran et al. 2002; Ueno et al. 2009; Breitz et al. 2003a)
	EDTMP	(Sohaib et al. 2011; Bahrami-Samani et al. 2010; Appelbaum et al. 1992; Louw et al. 1996)
	PAM	(Vaez-Tehrani et al. 2016)
	TTHMP	(Yousefnia et al. 2014)
	APDDMP	(Marques et al. 2006; Zeevaart et al. 2001)
Antibodies	DO3A-4B4	(Ballard et al. 2011)
	CHX-A"DTPA-6D2	(Thompson et al. 2014)
	DOTA-CC49 MeO-DOTA-CC49	(Mohsin et al. 2006; Mohsin et al. 2011)
Other complexes	DTPA	(Majali et al. 2001; Hong et al. 2002)
	DOTA	(Das et al. 2003)
	Chitosan	(Ha et al. 2013; Huh et al. 2005; Kim et al. 2006; Kwak et al. 2005; Suzuki et al. 1998; Lee et al. 2006; Cho et al. 2010; Song et al. 2001; Lee et al. 2003)
	Oxine lipiodol	(Das et al. 2009b)
	PMMA	(Hirsch et al. 2008)
Microparticles	Glass	(Costa et al. 2009; Brown et al. 1991)
	Resin	(Turner et al. 1994; Subramanian et al. 2018; Costa and Osso Junior 2008)
	Alginate	(Zielhuis et al. 2007)
	Lipiodol-alginate	(Oerlemans et al. 2015)
	AcAc-PLLA	(Nijsen et al. 1999; Mumper et al. 1992)
	Polyester	(Mumper and Jay 1992)
	AcAc	(Bult et al. 2012; Bult et al. 2009)
	PO_4_	(Bult et al. 2012; Arranja et al. 2018)
	Hydroxiapatite	(Das et al. 2009a; Unni et al. 2002)
	Ferric hydroxide (FHMA)	(Makela et al. 2004; Makela et al. 2003b; Vuorela et al. 2005; Kraft et al. 2007; Cho et al. 2010; Makela et al. 2004; Ofluoglu et al. 2002)
Nanoparticles	Mesoroporous silica nanoparticles	(Di Pasqua et al. 2013)
	Mesoroporous carbon nanoparticles	(Kim et al. 2017)
	AcAc-DSPE-PEG	(Di Pasqua et al. 2012)
	AcAc-PLLA	(Hamoudeh et al. 2008)
	AcAc	(Bult et al. 2010)
Liposomes	DPPC:Chol:PEG-DSPE	(Zielhuis et al. 2006)
Patches	Tape	(Lee et al. 1997; Chung et al. 2000)
	Nanofibers	(Munaweera et al. 2014)
Ceramic materials	Seeds	(Diniz et al. 2017; Valente et al. 2011; Nogueira and Campos 2011; Nogueira and de Campos 2012; Nogueira and Campos 2016; Hosseini et al. 2013; Valente and Campos 2010; Won et al. 2005)
	Membranes	(Nogueira and de Campos 2012)
Generator	DTPA complex	(Smith et al. 1995)
	Macroaggregates	(Makela et al. 2003a; Park et al. 1996; McLaren et al. 1990; Sledge et al. 1977)
	DTPA-Biotin	(Ferro-Flores et al. 2004; Ferro-Flores et al. 2003)
	EDTMP	(Pedraza-Lopez et al. 2004a)
	MOFDOTMP	(Vosoghi et al. 2016)
	Chitosan microspheres	(Cho and Choi 2018)

**Table 2:** Clinical studies published at www.clinicaltrials.gov in which a compound with holmium-166 is used

Study title	Device/ drug	Tumour type	Compound	Sponsor	Time path	ClinicalTrials.gov Identifier	Reference(s)
QuiremSpheres Observational Study	device	Liver cancer, non-resectable tumors	Holmium-166 polylactic microspheres	Terumo Europe N.V.	2018 - (recruiting)	NCT03563274	
Holmium Radioembolization as Adjuvant Treatment to RFA for Early Stage HCC: Dose Finding Study	device	hepatocellular carcinoma	RFA + Holmium-166 polylactic microspheres	Leiden University Medical Center The Netherlands	2018 - (recruiting)	NCT03437382	
HEPAR Primary: Holmium-166-radioembolization in Hepatocellular Carcinoma Patients	device	hepatocellular carcinoma	SIRT Holmium-166 PLLA microspheres	Erasmus Medical Center Rotterdam, University Medical Center Utrecht The Netherlands	2017 - (recruiting)	NCT03379844	
Feasibility of Holmium-166 Micro Brachytherapy in Head and Neck Tumors	device	head-and-neck neoplasms	SIRT Holmium-166 PLLA microspheres	University Medical Center Utrecht The Netherlands	2016–2018 (terminated, slow accrual)	NCT02975739	
Holmium-166-radioembolization in NET After Lutetium-177-dotatate; an Efficacy Study	device	neuroendocrine tumours in the liver	SIRT Holmium-166 PLLA microspheres	University Medical Center Utrecht, The Netherlands	2016 - (recruiting)	NCT02067988	(Braat et al. 2018b; van Nierop et al. 2018)
Surefire Infusion System vs. Standard Microcatheter Use During Holmium-166 Radioembolization	device	colorectal metastases in the liver	SIRT Holmium-166-PLLA microspheres	University Medical Center Utrecht The Netherlands	2016 - (recruiting)	NCT02208804	(van Nierop et al. 2018; van den Hoven et al. 2016)
Radioactive Holmium Microspheres for the Treatment of Unresectable Liver Metastases	device	liver neoplasms	SIRT Holmium-166-PLLA microspheres	University Medical Center Utrecht The Netherlands	2012–2015 (completed)	NCT01612325	(Prince et al. 2018; Braat et al. 2018a; Prince et al. 2015; van Nierop et al. 2018)
Radioactive Holmium Microspheres for the Treatment of Liver Metastases	device	liver metastases liver tumours	SIRT Holmium-166-PLLA microspheres	University Medical Center Utrecht Utrecht, Netherlands	2009–2012 (completed)	NCT01031784	(Smits et al. 2012; van de Maat et al. 2013; Smits et al. 2013; Smits et al. 2010; Prince et al. 2014; Elschot et al. 2014; Braat et al. 2018a; van Nierop et al. 2018)
Radiation Therapy Using Holmium Ho 166 DOTMP Plus Melphalan and Peripheral Stem Cell Transplantation in Treating Patients With Multiple Myeloma	drug	multiple myeloma and plasma cell neoplasm	melphalan + Holmium-166- DOTMP	Fred Hutchinson Cancer Research CenterSeattle, Washington, United States	2004–2010 (completed)	NCT00004158	
Melphalan With or Without Holmium Ho 166 DOTMP Followed by Peripheral Stem Cell Transplantation in Treating Patients With Multiple Myeloma	drug	multiple myeloma and plasma cell neoplasm	melphalan + Holmium-166- DOTMP	Fred Hutchinson Cancer Research Center Seattle, Washington, United States	2004–2010 (completed)	NCT00008229	
Study Comparing STR (Skeletal Targeted Radiotherapy) Plus Melphalan to Melphalan Alone, With Stem Cell Transplant in Multiple Myeloma	drug	multiple myeloma	Holmium-166- DOTMP	Poniard Pharmaceuticals	2004–2009 (terminated)	NCT00083564	(Giralt et al. 2003; Breitz et al. 2003b)
Holmium Ho 166 DOTMP Followed by Peripheral Stem Cell Transplantation in Treating Patients With Metastatic Ewing's Sarcoma or Rhabdomyosarcoma That Has Spread to the Bone	drug	metastatic cancer, sarcoma	Holmium-166- DOTMP	Fred Hutchinson Cancer Research Center Seattle, Washington, United State	2003–2011 (completed)	NCT00006234	
Chemotherapy, Holmium Ho 166 DOTMP, and Peripheral Stem Cell Transplantation in Treating Patients With Multiple Myeloma	drug	multiple myeloma and plasma cell neoplasm	Holmium-166- DOTMP	Poniard Pharmaceuticals	2003–2009 (completed)	NCT00045136	
A Trial of Skeletal Targeted Radiotherapy Using Holmium-166-DOTMP in Patients With Multiple Myeloma	drug	multiple myeloma	Holmium-166-DOTMP	Poniard Pharmaceuticals	2002–2009 (terminated, business reasons)	NCT00039754	

## Intratumoural applications

### Chitosan

The medical isotope ^166^Ho is gaining more and more interest from nuclear physicians in the treatment of tumours. Both drugs and medical devices labelled with radioactive ^166^Ho are used for internal radiotherapy. One of the therapy possibilities is the direct intratumoural treatment, in which the radioactive compound is injected with a needle directly into the tumour (Fig. 3). Such an intratumoural application can be found in for instance the ^166^Ho-labelled chitosan complex, which has been studied in different types of tumours such as brain (Ha et al. 2013; Huh et al. 2005), liver (Sohn et al. 2009; Kim et al. 2006) and prostate cancer (Kwak et al. 2005). Chitosan is a polymer obtained from the deacetylation of chitin which can form chelates with heavy metals (Suzuki et al. 1998; Park 1997). Its solubility in water is pH-dependent. Below pH 4.0, it is readily soluble in water. However, under neutral or basic conditions, it is converted into a gelatinized material enabling the retention of the complex at the administration site. The ^166^Ho-labelled chitosan complex was developed as a radiopharmaceutical for cancer therapy by the Korean Atomic Energy Research Institute. Preparation of this complex for injection involves vigorously mixing a solution composed of [^166^Ho]Ho nitrate and chitosan for 2 to 3 min (Sohn et al. 2009; Kim et al. 2006; Kwak et al. 2005). A retention of ^166^Ho at the injection site of around 90% has been demonstrated using the ^166^Ho-labelled chitosan complex due to the formation of a gel in the microenvironment of the injection site (Kwak et al. 2005; Suzuki et al. 1998; Park 1997; Muzzarelli et al. 1988). In animal studies, it was shown that a significant decrease in tumour volume was measured in the treated group compared to the control group (Huh et al. 2005; Kwak et al. 2005; Suzuki et al. 1998). In clinical research, positive effects have been observed as well: Kim et al. demonstrated complete tumour necrosis in 80.8% (42 of 52) of the patients with small hepatocellular carcinoma (Kim et al. 2006). In a study of Ha et al., 70% of the patients with recurrent cystic brain tumours responded positively to treatment with ^166^Ho-labelled chitosan, without systematic absorption or leakage (Ha et al. 2013; Huh et al. 2005).

^166^Ho-labelled chitosan has also been studied in order to treat renal cysts. Seventeen patients with renal cysts were injected with ^166^Ho-labelled chitosan under ultrasonographic guidance. At the end of the follow-up, 90% of the treated cysts had underwent a complete or near complete regression (Kim et al. 2004).

### Ceramic materials

Another type of intratumoural treatment is brachytherapy (Fig. 3), in which radioactive ceramic materials are implanted in the tumour. These devices can form a variety of shapes and sizes and have been widely used because they can deliver a high local dose to the tumour while the surrounding tissue is spared, and they have shown to be biocompatible and biodegradable (Roberto et al. 2003; Campos et al. 2008). They are usually synthesized by the sol-gel technique which is a chemical method using temperatures below those used in traditional methods to process glass and ceramics (Hench and West 1990). This technique involves the homogenization of several components, pouring into moulds and then following a specific procedure of gelation, aging, drying and heat treatment (Hench and West 1990). The preparation of seeds (Diniz et al. 2017; Valente et al. 2011; Nogueira and Campos 2011; Nogueira and de Campos 2012; Nogueira and Campos 2016) and membranes (Nogueira and de Campos 2012) composed of ^165^Ho have been reported in the literature. Seeds usually have dimensions ranging from 0.3 to 0.8 mm in diameter and 1.5 to 1.8 mm in length and a density of 2 to 3.7 g/cm^3^ (Valente et al. 2011; Nogueira and Campos 2011). Holmium concentrations of 20 wt% (Valente et al. 2011) up to 30 wt% (Nogueira and Campos 2016) are achieved in these materials and it has been shown that the radionuclides are homogeneously distributed in the seeds matrix (Nogueira and Campos 2011). Neutron activation is performed on the final material and, although different elements in the matrix can be neutron activated, it has been shown that the radionuclide impurities are negligible due to low neutron cross-section of the materials used in their fabrication (Nogueira and Campos 2011). Insertion of other elements in the holmium-based ceramic materials such as barium (Nogueira and de Campos 2012) and zirconium (Nogueira and Campos 2016) to improve the radiological contrast allowing visualization of the seeds using conventional X-rays and mammography has also been investigated.

^166^Ho –labelled ceramic seeds have been studied in brain tissue in an animal study and also in a simulation study in human breast tissue (Diniz et al. 2017; de Campos et al. 2016). The advantages of the ^166^Ho-labelled seeds are the high dose rate and not having to remove the seeds after the treatment. Diniz et al. investigated the safety of degradable seeds in rat brains, using non-neutron activated seeds. The results showed no neurological or brain architectural changes, indicating a safe procedure (Diniz et al. 2017). In a study on brachytherapy in breast cancer by de Campos et al. a computational simulation was performed to provide a dosimetric analysis of temporary ^192^Ir-labelled seeds compared to permanent ^166^Ho-labelled seeds. It was shown that the spatial dose distribution of ^166^Ho was confined to the implanted volume, whereas this is not the case for brachytherapy using iridium-192, which is frequently used (de Campos et al. 2016). Monte Carlo simulations (Hosseini et al. 2013) and MIRD methodology (Valente and Campos 2010) have been used to determine the range and total absorbed dose of ^166^Ho-labelled ceramic seeds. The results were compared with seeds containing ^32^P, ^90^Y (Hosseini et al. 2013) or samarium-132 (^132^Sm). The radial dose values of the ^166^Ho-labelled seeds were concluded to be higher than other seeds at distances smaller than 5 mm (Hosseini et al. 2013; Valente and Campos 2010).

### Microspheres

^166^Ho-labelled microspheres (^166^Ho-MS) have been developed for SIRT (also called radioembolization) (see paragraph on Selective internal radiation therapy) (Nijsen et al. 1999; Nijsen et al. 2004; Nijsen et al. 2001), but may potentially be used in microbrachytherapy (Bakker et al. 2017) for treatment of liver tumours (Bult et al. 2013a), kidney tumours (Bult et al. 2012) and head-and-neck tumours (van Nimwegen et al. 2018; Bakker et al. 2018). Moreover, this intratumoural approach can be used also for other tumour types such as pancreas tumours and even lung tumours. The intratumoural potential of ^166^Ho-MS has been investigated in both veterinary and human patients (Bakker et al. 2018; Bult et al. 2013a; van Nimwegen et al. 2018; Bult et al. 2013b; Van de Bovenkamp et al. 2009). Currently, two types of ^166^Ho-MS for intratumoural application, i.e., ^166^Ho-labelled acetylacetonate microspheres (^166^Ho-AcAc-MS) and ^166^Ho-labelled poly(L-lactic acid) microspheres (^166^Ho-PLLA-MS) were tested in animals. The ^166^Ho-AcAc-MS are prepared by a conventional oil in water (o/w) emulsification and solvent evaporation method by dissolving ^165^Ho-labelled AcAc crystals in chloroform and adding this organic solution to an aqueous phase containing an emulsifier. The emulsion formed is stirred until complete evaporation of the chloroform. The method enables preparation of microspheres with different sizes by varying for instance the stirring speed (Bult et al. 2012; Bult et al. 2009). The ^166^Ho-PLLA-MS are prepared using a similar method with the exception that the polymer (PLLA) is added to the organic solution before emulsification (Nijsen et al. 1999). After preparation, both microspheres have to be neutron activated to obtain radioactive ^166^Ho-AcAc-MS and ^166^Ho-PLLA-MS which can later be used for intratumoural treatments. In 2013, the first two papers on ^166^Ho-AcAc-MS in microbrachytherapy were published by Bult et al. (Bult et al. 2013a; Bult et al. 2013b). In the first study, the feasibility of applying ^166^Ho-AcAc-MS as an intratumoural treatment for renal tumours was demonstrated (Bult et al. 2013b). In the second study three domestic cats with spontaneous liver cancer were treated by administration of ^166^Ho-AcAc-MS via ultrasound guided percutaneous injections. In all cats the treatment was well tolerated and life was extended with good quality of life (Bult et al. 2013a). Van Nimwegen et al. treated 13 cats with inoperable squamous cell carcinoma by intratumoural injection of ^166^Ho-PLLA-MS. A response rate of 55% was observed with minimal side effects (van Nimwegen et al. 2018). Bakker et al. used ^166^Ho-PLLA-MS as a palliative treatment for patients with recurrent head-and-neck squamous cell carcinoma. Due to technical difficulties, a low patient dose and non-homogeneous distribution was observed which is probably the reason that a relatively low therapeutic efficacy was seen, however in none of the cases adverse effects were observed (Bakker et al. 2018).

### Holmium solutions

The possibility of using dissolved ^166^Ho has also been studied. Suzuki et al. injected dissolved ^166^Ho salts into the liver of a male rat, resulting in a high uptake in blood and other tissue, however there was no retention of ^166^Ho at the injection site (Suzuki et al. 1998). In a mouse model of melanoma, free ^166^Ho was injected into the tumours with one single injection. The injection fluid was not always uniformly distributed throughout the tumours causing tumour regrowth. It was suggested that free ^166^Ho may be used for solid and firm tumours, although hypervascularized tumours may not retain the soluble form (Lee et al. 2002). Tumour growth was increasingly impaired when intratumoural injection of free ^166^Ho was combined with the injection of dendritic cells 1 week after ^166^Ho injection, compared to ^166^Ho injection alone. Nineteen days after ^166^Ho injection, the average tumour size in the ^166^Ho injection alone group was 1658 mm^2^, against 444 mm^2^ in the ^166^Ho and dendritic cell group (Lee et al. 2006).

## Intravenous applications

### Antibodies and peptides

Targeted therapy with radionuclides is an upcoming form of cancer treatment, in which radioisotopes are labelled to antibodies or other tumour-seeking peptides (Fig. 3). Different antibodies (Table 1) have been conjugated to ^166^Ho. For this, (monoclonal) antibodies (mAbs) are first conjugated to an appropriate chelator such as DO3A-4B4 (Ballard et al. 2011), DOTA, methoxy-DOTA (MeO-DOTA) (Mohsin et al. 2006) or CHX-A" DTPA (DTPA = diethylenetriaminepentaacetic acid) (Thompson et al. 2014). Radiolabeling with ^166^Ho is then performed in an appropriate buffer by mixing the antibody-chelator conjugate with the radioactive ^166^Ho dissolved in hydrochloric acid. The complex is then purified typically by size exclusion chromatography (Mohsin et al. 2006).

Melanoma is an example of a disease in which labelled antibodies or peptides with ^166^Ho have been used as a treatment. Ballard et al. labelled a 4B4 peptide with three types of isotopes, i.e. ^166^Ho, ^177^Lu and samarium-153 (^153^Sm), as a potential therapy for melanoma. In vitro binding assays demonstrated a lower binding efficiency and specific activity of ^166^Ho and ^153^Sm compared to ^177^Lu, as a result ^166^Ho and ^153^Sm have not been included in further in vivo experiments (Ballard et al. 2011). In a study by Thompson et al., a comparison was made between ^166^Ho-, ^90^Y-, and ^188^Re-labelled 6D2 mAbs as a potential treatment for melanoma. The results showed a comparable therapeutic effect between ^166^Ho- and ^188^Re-labelled 6D2 mAbs after injection of an activity of 37 MBq. Additionally, no toxic effect was observed as a result of injection of ^166^Ho or ^188^Re complex, whereas the ^90^Y complex was toxic to mice and did not produce an antitumour effect (Thompson et al. 2014).

Over the years, a strong interest in targeted therapies for colorectal cancer has been developed. In a preclinical study, mice were intravenously injected with ^166^Ho-, promethium-149 (^149^Pm)- or ^177^Lu-labelled CC49 mAbs 14 days after implantation of human colon tumours. Biodistribution results showed a maximum tumour uptake of ^166^Ho at 96 h post injection (Mohsin et al. 2006), which is rather late. Since the half-life of ^166^Ho is only 26.8 h, the therapeutic effect may then be limited. In another study the same mAbs and radionuclides were compared and the ^166^Ho-labelled mAbs yielded the least favourable results in terms of tumour doubling time and survival (Mohsin et al. 2011). These unfavourable results may also be the result of the combination of short half-life (26.8 h) and late (96 h) maximum tumour uptake, as the ^166^Ho may already be decayed when arriving to the tumour. Khorami-Moghada et al. provided a study on a colon cancer model in mice, labelling ^166^Ho to the VEGF-A antibody bevacizumab. The biodistribution results show high uptake in the liver, blood, kidneys and in the tumours (Khorami-Moghadam et al. 2013). Unfortunately, the article is not very clear in its material and methods and therefore it is difficult to estimate the real value.

### Bone-seeking agents

Bone marrow transplantation poses a potentially curative treatment to various hematologic malignancies. Prior to the autologous or allogeneic transplantation high local radiation doses are needed to destroy the old bone marrow. High doses generated by external radiation may cause damage to other organs, which is unfavourable. Therefore, bone seeking radiopharmaceuticals have been developed to generate a high local dose and limiting dose to other tissues.

An efficient and safe radiopharmaceutical for bone marrow ablation requires the formation of a stable complex between the bone-seeking agent and the radionuclide. Phosphonate chelates are commonly used for this purpose due to their excellent specificity for bone localization. When combined with radioactive isotopes, the complexes can deliver high levels of radiation to bone and bone marrow, leaving normal tissues unaffected. The isotope ^166^Ho has been conjugated to several bone-seeking agents for bone marrow ablation such as EDTMP (ethylene-diamine-tetramethylene phosphonic acid) (Sohaib et al. 2011; Bahrami-Samani et al. 2010; Appelbaum et al. 1992; Louw et al. 1996), DOTMP (Bayouth et al. 1995a; Bayouth et al. 1995b; Parks et al. 1993; Giralt et al. 2003; Breitz et al. 2006; Rajendran et al. 2002; Ueno et al. 2009; Breitz et al. 2003a), TTHMP (triethylene tetramine hexa (methylene phos- phonic acid)) (Yousefnia et al. 2014), PAM (pamidronate) (Vaez-Tehrani et al. 2016), APDDMP (N,N-dimethylenephosphonate-1-hydroxy-4-aminopropylidene-diphosphonate) (Marques et al. 2006; Zeevaart et al. 2001), among others. The preparation of the ^166^Ho-radiolabelled complexes is performed very similarly with the different bone-seeking agents. In general, a target of holmium oxide is neutron activated which is afterwards dissolved in hydrochloric acid to form ^166^HoCl_3_. Then, a solution containing the dissolved chelator complex (e.g. DOTMP) is added and the pH is adjusted to 7–8. Complexation occurs within typically 1 h at room temperature with high radiochemical purity (more than 99%) (Sohaib et al. 2011; Bahrami-Samani et al. 2010; Bayouth et al. 1995a; Yousefnia et al. 2014).

Appelbaum et al. showed that after 24 h post injection of the bone seeking phosphonate EDTMP labelled with ^166^Ho, the [^166^Ho]Ho EDTMP concentration was 200-fold higher in bone than in other organs (Appelbaum et al. 1992). The results of a study performed by Bahrami-Samani et al. in wild-type rats demonstrated significant bone accumulation (> 70%) of [^166^Ho]Ho EDTMP after 48 h (Bahrami-Samani et al. 2010). However, in a study by Louw et al., [^166^Ho]Ho EDTMP turned out to be significantly inferior to [^153^Sm]Sm EDTMP in terms of pharmacokinetics, biodistribution and skeletal localization (Louw et al. 1996). These results differ considerably from the results found by Appelbaum et al. (Appelbaum et al. 1992). The discrepancy might, among others, be due to the use of S-values for human children by Appelbaum et al., whereas adult human S-values were used by Louw et al. (Louw et al. 1996). Sohaib et al. also demonstrated inferiority of [^166^Ho]Ho EDTMP compared to [^90^Y]Y EDTMP, with a skeletal accumulation of only approximately 27% (46% in [^90^Y]Y EDTMP) (Sohaib et al. 2011).

Pedraza-Lopez et al. have labelled ^166^Dy/^166^Ho with the bone seeker EDTMP to treat haematological malignancies. ^166^Dy_2_O_3_ was neutron activated and converted to ^166^DyCl_3_ which was then added to a solution containing EDTMP in phosphate buffer (pH 8.0). A radiochemical purity of 99.3 ± 0.6% was achieved. Animal studies showed a fast blood clearance of [^166^Dy]Dy/[^166^Ho]Ho EDTMP and a skeletal uptake of 22.32 ± 1.86% ID/g at 2 h and 20.12 ± 1.94% ID/g after 10 days. Theoretical bone marrow absorbed dose calculations indicate that the [^166^Dy]Dy/[^166^Ho]Ho EDTMP in vivo generator system produced 3.47 times more dose than [^166^Ho]Ho DOTMP per unit of initial activity in the skeleton (Pedraza-Lopez et al. 2004a). The same group also demonstrated that the [^166^Dy]Dy/[^166^Ho]Ho EDTMP system induced considerable cytotoxicity, genotoxicity and severe myelosuppression in mice at bone marrow absorbed doses of 18–23 Gy, suggesting that this system could potentially be a good agent for use in humans (Pedraza-Lopez et al. 2004b).

In an animal study on splenectomized young adult beagle dogs, a complete ablation of hematopoietic marrow was seen within 7 days after receiving a radiopharmaceutical dosage of 370 MBq/kg body weight of [^166^Ho]Ho DOTMP (Parks et al. 1993). The pharmacokinetics, biodistribution and absorbed dose estimation of the [^166^Ho]Ho DOTMP complex has also been studied in six patients suffering from multiple myeloma. A high uptake in the skeleton and rapid clearance from the blood was observed (Bayouth et al. 1995a). Two larger phase I/II dose escalation studies in human patients showed a fast bone uptake, rapid clearance from the blood, no retention in soft tissue and minimal systemic toxicity (Giralt et al. 2003; Rajendran et al. 2002). A therapeutic dose of [^166^Ho]Ho DOTMP was given to 83 patients with multiple myeloma. In 35% of the patients complete remission was achieved and no acute toxicity issues were seen (Giralt et al. 2003). In this cohort study, dosimetry and toxicity were also assessed. Doses in the marrow, bladder and kidney range from 13 to 59 Gy, 4.7 to 157 Gy, and 0.5 to 7.9 Gy, respectively. Hemorrhagic cystitis was observed in a number of patients who received a dose of more than 40 Gy to the bladder wall. Renal toxicity related to [^166^Ho]Ho DOTMP was observed in 7 patients (12%) (Breitz et al. 2003a). Ueno et al. treated 6 women with bone-only metastasized breast cancer with [^166^Ho]Ho DOTMP. After a follow-up time of 6 years, 2 of the 6 patients sustained complete response. An acceptable toxicity profile was also described in this paper (Ueno et al. 2009). Since the results are very promising, five clinical trials have been performed on [^166^Ho]Ho DOTMP, of which no papers have been published yet, with the following clinicaltrials.gov identifier numbers: NCT00045136, NCT00004158, NCT00008229, NCT00039754, NCT00006234 (Table 2).

Vosoghi et al. developed a highly-stable metallic organic framework (MOF) as a bone seeking agent composed of DOTMP, ^166^Dy/^166^Ho generator and CuCl_2_. The MOF was formed by stirring neutron activated [^166^Dy]Dy/[^166^Ho]Ho nitrate, CuCl_2_ and DOTMP at room temperature. Purification of the ^166^Dy from the ^166^Ho and other isotopes was achieved by chromatography before preparation of the MOF. The particles obtained had a size ranging from 60 to 100 nm. The product has shown to have high affinity for simulated bone matrix and comparable to that of the FDA-approved radiopharmaceutical Quadramet ([^153^Sm]Sm EDTMP) (Vosoghi et al. 2016).

Two other bone-seeking agents are nano-hydroxyapatite dopped with ^166^Ho (da Silva et al. 2017) and [^166^Ho]Ho PDTMP (Zolghadri et al. 2013). In both studies, a significant bone uptake was demonstrated, but no further research has been done.

### Nanoparticles

Nanoparticles containing holmium have been used for different applications with different compositions (Table 1). Munaweera et al. developed magnetic nanoparticles containing both ^166^Ho and a platinum-based chemotherapeutic to treat lung cancer. This study demonstrated that the nanoparticles are toxic to the tumour cells and that tumour to liver ratios increases when an external magnetic field was applied (Munaweera et al. 2015). Zielhuis et al. prepared nanosized liposomes (~ 120 nm) using a conventional thin-film hydration technique and loaded the liposomes with the radioactive [^166^Ho]Ho DTPA-lipid complex which was incorporated in the liposomal bilayer. The high stability and paramagnetic properties make them suitable for treatment and multimodal imaging with SPECT and MRI (Zielhuis et al. 2006).

## Selective internal radiation therapy

Selective internal radiation therapy (SIRT, also called radioembolization) is a form of internal radiation therapy, during which millions of microspheres are administered into an artery, close to a target lesion. These microspheres of around 30 μm in diameter are administered through a catheter and carried by the blood flow until they lodge at the arteriolar level. Clinically, SIRT is applied as novel treatment for liver malignancies. The rationale is that liver tumours mainly depend on the hepatic artery for their blood supply, whereas the healthy liver tissue is mainly supplied by the portal vein. Therefore, microspheres in the hepatic artery will especially target liver tumours. This will result in a higher radiation dose to the liver tumours, whilst sparing the healthy liver tissue (Nijsen et al. 2002a).

Currently, the only commercially available and clinically used microspheres for SIRT based on the isotope ^166^Ho are ^166^Ho-PLLA-MS (QuiremSpheres, Quirem Medical, the Netherlands). Other microspheres with ^166^Ho that were investigated for application within SIRT procedures are MS-PDLLA-HoAcAc (Nayak and Lahiri 1999) and resin-based microspheres from Turner et al. (Turner et al. 1994; Zielhuis et al. 2007; Mumper and Jay 1992). The polymer-based microspheres used for SIRT are typically prepared by the emulsification and solvent evaporation method (see section 3.3) and have the advantage of near blood plasma density and biocompatibility (Nijsen et al. 2002a; Mumper et al. 1992). However, polymers are usually sensitive to neutron irradiation which can lead to damage of the microspheres after long neutron activation (Nijsen et al. 1999; Nijsen et al. 2002b). Nevertheless, the ^166^Ho-PLLA-MS microspheres can be neutron activated till high amounts of activity, sufficient for transportation to the hospital and clinical application (Vente et al. 2009). Resin-based microspheres present the advantage of easier preparation performed through the incubation of the radioactive ^166^Ho with commercially available resins. Bio-Rex resins which are made of an acrylic polymer with carboxylic groups were used to bind ^166^Ho with high-yield (94.53% at pH 8.5) by Subramanian et al. (Subramanian et al. 2018). Aminex A-5 resin, based on styrene divinylbenzene copolymer with sulphonic acid, has also been conjugated to ^166^Ho by Turner et al. (Turner et al. 1994). Alginate microspheres can also be loaded with ^166^Ho after neutron activation (Das et al. 2009a). Last, chitosan microspheres containing the ^166^Dy/^166^Ho in vivo generator have also been prepared. Stability studies showed that 5–10% of the radioactivity was released in human serum (Cho and Choi 2018).

### Imaging (SPECT, MRI and CT)

Smits et al. described that the activity for using holmium microspheres in SIRT can be calculated with the following formula: A_Ho166_ (MBq) = Liver dose × 63 (MBq/J) × LW (kg) where A_Ho166_ is the administered activity, LW is the tissue weight, and dose is the intended absorbed radiation dose in Gy (Smits et al. 2012; van de Maat et al. 2013). When imaging the ^166^Ho distribution with SPECT, medium-energy collimators are often used with energy windows of 80.6 keV (15% window width) for the ^166^Ho photopeak and 118 keV (12%) for correction for down-scattered high-energy photons. A typical protocol will be 120 projections of 30 s orbits around the liver. Data for the distribution of ^166^Ho-PLLA-MS deposition in liver SIRT treatments have been reconstructed to a 128 · 128 · 128 matrix with an isotropic voxel size of 4.7 mm or 4.8 mm using an ordered-subsets expectation maximization algorithm including resolution recovery and a hybrid method for scatter and attenuation correction (Elschot et al. 2011; Smits et al. 2013).

The holmium in the microspheres can be used for quantitative MR imaging, since it is a highly paramagnetic metal. The imaging is usually performed on clinical whole-body MR systems of 1.5 or 3 T (Smits et al. 2013). For quantitative measurements of the ^166^Ho-microsphere biodistribution, a multi-slice multi-gradient echo sequence is used, sampling the MR imaging signal of the free induction decay. The holmium quantification is performed though T2*-weighted turbo spin echo imaging (Seevinck et al. 2007; van de Maat et al. 2013; Smits et al. 2013; Seevinck et al. 2012).

Agglomerations of holmium microspheres can be visualized through CT imaging, in practice especially holmium microspheres that contain relatively high amounts of holmium. If these microspheres are concentrated, which is the case in intratumoural injections, CT can be used for quantitative detection (Seevinck et al. 2007; Bakker et al. 2018). Moreover, novel microspheres with a much higher holmium content are currently developed (Bult et al. 2009; Arranja et al. 2018). These microspheres are expected to be visible on CT in even lower concentrations.

### Head-and-neck tumours

Van Es et al. performed two animal studies in which Vx2 cells were subcutaneously injected into the auricles of rabbits. After reaching a tumour size of 4 cm^2^, ^165/166^Ho-PLLA-MS (van Es et al. 2001; Van Es et al. 2001) or ^99m^Tc-labelled Dextran hydrogel (Dex) MS (van Es et al. 2001) were injected into the caudal auricular artery. In the first study a comparison was made between injecting ^165^Ho-PLLA-MS and ^166^Ho-PLLA-MS. Complete remission of 79% and 86% was obtained by injecting ^165^Ho-PLLA-MS and ^166^Ho-PLLA-MS, respectively. Although there was a high remission rate, the limited additional effect of irradiation might be explained by the high sensitivity of the Vx2 tumour model to vascular occlusion. In addition, retention of approximately 40% of the MS into the infusion system may have led to an ineffective dosage (Van Es et al. 2001). In the second study, injection of ^166^Ho-PLLA-MS was compared to Dex MS. 51% of the ^166^Ho-PLLA-MS, with a diameter of 19 μm, shunted to the lungs, whereas 95% of the Dex MS were retained in the tumour (van Es et al. 2001).

### Liver tumours

In a biodistribution study in which SIRT was performed with ^166^Ho-PLLA-MS on rats with implanted liver tumours, the radioactivity in the tumours was six time higher than in the non-targeted liver tissue, indicating the ^166^Ho-PLLA-MS deposition was confined to the liver (Nijsen et al. 2001). In order to assess the clinical effects of the microspheres, healthy pigs received an activity up to 6.5 GBq through the liver artery, which corresponds with an estimated absorbed liver dose of 120 Gy. After 2 months of follow-up, it was concluded that the toxicity profile of holmium microspheres is low and that administration of doses up to 100 Gy is not associated with clinically relevant side effects (Vente et al. 2008).

Seppenwoolde and his colleagues demonstrated the feasibility of fully MR-guided SIRT with holmium microspheres in pigs. Whereas the positioning of the catheter normally takes place under X-ray guidance, in this study a passive tracking sequence was used to visualize paramagnetic markers on the catheter. Although some drawbacks are described (such as difficulty in visualization of the catheter due to respiratory motion artefacts) this article is the first to report on real-time imaging of holmium microspheres in an anthropomorphic in vivo model as a proof of principle that real-time image guided treatment is possible under MRI (Seppenwoolde et al. 2005).

**Fig. 4:**
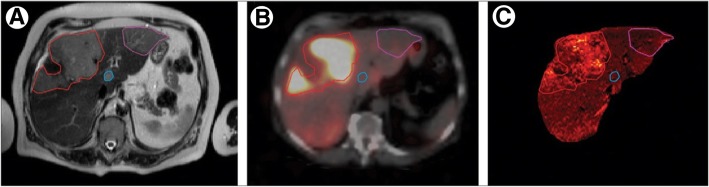
Intrahepatic visualisation of ^166^Ho-microspheres after SIRT. T2-weighted MRI of the liver in a patient with several ocular melanoma liver metastases, outlined by coloured regions of interest (**a**). After SIRT, the distribution of ^166^Ho-PLLA-microspheres within the liver was visualized by single-photon-emission CT (**b**) and R2-weighted MRI (**c**). Reprinted from The Lancet Oncology, Vol. 13, Smits et al., Holmium-166 SIRT in patients with unresectable, chemorefractory liver metastases (HEPAR trial): a phase 1, dose-escalation study, 1025–1034, Copyright 2012, with permission from Elsevier

The first clinical trial in which ^166^Ho-PLLA-MS were administered to patients was the phase 1 HEPAR 1 trial (2009–2011) (Smits et al. 2012; Smits et al. 2010) (Table 2). In this study, 15 patients with liver metastases of any origin were treated by SIRT at different whole-liver absorbed dose levels ranging from 20 to 80 Gy (Fig. 4). In the 80 Gy cohort, dose-limiting toxicity occurred in two out of three patients. Therefore, the maximum tolerated radiation dose was identified as 60 Gy. Exposure rates (μSv/h) from patients were measured at 1.0 m distance from a lateral and frontal position at 0, 3, 6, 24, and 48 h after infusion. The total effective dose equivalent to a contact of patients treated with ^166^Ho-labelled MS did not exceed the NRC limit of 5 mSv. Contact restrictions 6 h after treatment are unnecessary for infused activities < 7 GBq (Prince et al. 2014).

After SIRT of liver metastases with ^166^Ho-PLLA-MS was deemed safe in the HEPAR 1 trial, the HEPAR 2 trial was performed to investigate the efficacy of the ^166^Ho SIRT (Prince et al. 2018) (Table 2). In this trial, 38 patients with liver metastases refractory to systemic therapy and ineligible for surgical resection were treated with ^166^Ho-PLLA-MS. In 73% of the patients, the target lesions showed complete response, partial response, or stable disease (disease control) at 3 months. There was an acceptable toxicity profile. The most common adverse events during follow-up were gastrointestinal complaints, i.e. nausea, abdominal pain and fatigue. In a subgroup analysis on the patients with liver-dominant colorectal cancer metastases, a median survival of 13.4 months (95% CI, 8.2–15.7 mo) was seen, which is comparable to the reported range of 8.3–15.2 mo after ^90^Y SIRT (Prince et al. 2018).

In order to prevent any unintentional deposition of the microspheres in tissue other than the liver, a safety procedure is typically performed before the actual treatment. During this procedure, relevant extrahepatic arteries are coiled and a scout dose of technetium-99 m-labelled macro-aggregated albumin (^99m^Tc-MAA) is administered. SPECT-imaging is then performed in order to estimate the extrahepatic shunting of activity. In literature, it has been argued multiple times that this ^99m^Tc-MAA scout dose is not an accurate estimation of the actual microsphere distribution, due to morphological (different non-spherical shapes, large size distribution) and biochemical differences between ^99m^Tc-MAA and microspheres such as ^166^Ho-labelled MS and ^90^Y-labelled MS (Wondergem et al. 2013; Elschot et al. 2014).

For this matter, studies have been performed to investigate the possibility of using a scout dose of ^166^Ho-labelled MS (^166^Ho-SD) before treatment. Braat et al. (Braat et al. 2018a) used a scout dose (250 MBq) in 82 patients. In 6 patients there were extrahepatic depositions, however, no adverse events related to this were observed during a median follow-up of 4 months (range 1–12 months). In another study, the extrahepatic depositions of ^99m^Tc-MAA were objectified in 166 patients, and then the absorbed dose was calculated as if these patients had received a ^166^Ho-SD. Only in 2 cases the theoretical dose exceeded 50 Gy (Prince et al. 2015). These results support the safety of a 250 MBq ^166^Ho-SD in a clinical setting.

Radosa et al. investigated the clinical feasibility, technical success and toxicity of SIRT with ^166^Ho-labelled MS in 9 patients with HCC (Radosa et al. 2019). The median administered activity was 3.7 GBq. The treatment was found to be feasible and safe, with no significant hepatotoxicity, as 4 patients suffered from mild and transient post radioembolization syndrome and no indicators of SIRT induced liver disease (REILD) were observed. Eight patients showed a good response and only one patient had a progressive disease at 6 months follow-up. All in all, the results with holmium microspheres were deemed at least comparable to yttrium microspheres in terms of safety.

## Radiosynoviorthesis

### Ferric hydroxide macroaggregates

Besides cancer treatment, ^166^Ho may also be used as a radionuclide in a technique called radiosynoviorthesis to treat chronic synovitis or recurrent hemarthrosis in coagulation disorders. In this treatment, a ^166^Ho-labelled ferric hydroxide macroaggregates (FHMA) complex is injected into the affected joints (Fig. 3) to generate local irradiation. Various studies have been performed on this type of treatment. In a Monte Carlo simulation study by Ferro-Flores et al. it was shown that ^166^Ho generates a favourable radiation dose to the articular cartilage and bone surface (Ferro-Flores et al. 2004). In an animal study, 30 rabbits received intra-articular injections with ^166^Ho-labelled FHMA, resulting in acute focal radiation necrosis without hyperplasia of synoviocytes. Autoradiography showed an uneven distribution of the radiotherapeutical along the synovial lining. After 3 days, the majority of the ^166^Ho-labelled FHMA complex had leaked out of the joint or was phagocytized by the synoviocytes (Makela et al. 2002). In a study by Makela et al., six horses were treated with ^166^Ho-labelled FHMA, with a mean activity of 1000 MBq/joint. ^165^Ho-labelled FHMA was used as a control. In this study, acute focal radiation necrosis (of the synovium) was observed as well, but in contrast to the study on rabbits, no radiopharmaceutical leakage was found (Makela et al. 2003a). The articular cartilage of the horses showed only mild signs of degeneration as a result of this therapy (Makela et al. 2004). In another study, the short and long-term effects of radiosynoviorthesis with ^166^Ho-labelled FHMA were observed in mature and growing rabbits. The radiation effects in the growing rabbits included mild cartilage fibrillation and downregulation of cartilage-specific genes (Makela et al. 2003b). In a study by Vuorela et al., SPECT and MRI images of human patients treated with ^166^Ho-labelled FHMA were fused. SPECT images were used to localize the ^166^Ho, whereas the T1- weighted Gd-DTPA MR images were used to visualize the inflamed synovium. A higher uptake of ^166^Ho in regions associated with a higher level of synovitis was seen (Vuorela et al. 2005). Kraft et al. studied the therapeutic effect of ^166^Ho-labelled boron macroaggregates. In this study, 17 knees of 15 human patients with chronic synovitis were treated with a mean activity of 972 MBq. Only an insignificant leakage of radiopharmaceuticals was observed. Six months after treatment, 73% of the patients experienced a lower sense of pain. In addition, 2 patients had no knee exudation anymore and a decrease of knee exudation was seen in 4 patients (Kraft et al. 2007).

### Chitosan

Synovitis can also be treated with intra-articularly injected ^166^Ho-labelled chitosan. The preparation of the complex is similar to the one used for intratumoural application (section 3.1). In a study on human patients with rheumatoid arthritis, most of the injected radioactivity was localized within the injected cavity and limited radioactive excretion and negligible extra-articular leakage were observed (Cho et al. 2010; Song et al. 2001). Cho et al. stated that ^166^Ho-labelled chitosan is a favourable agent compared to the conventional treatment of synovititis using other radioisotopes, based on a higher permeability to soft tissue, smaller extra-articular leakage and higher amount of induced synovial necrosis (Cho et al. 2010). Lee et al. evaluated the response using MR imaging and found decreased joint effusion at 4 months after the treatment with ^166^Ho-labelled chitosan (Lee et al. 2003).

### Microspheres

Next to SIRT, ^166^Ho-PLLA-MS have also been studied as an agent for radionuclide synovectomy. In a study by Mumper et al., ^166^Ho-PLLA-MS were injected in the knee joints of six healthy rabbits. At 120 h post injection, intra-articular ^166^Ho retention of approximately 98% was found with no uptake in the lymph nodes (Mumper et al. 1992).

### ^166^Dy/^166^Ho macroaggregates

Several ^166^Dy/^166^Ho-labelled macroaggregates have also been prepared for radiation synovitis (Park et al. 1996; McLaren et al. 1990; Sledge et al. 1977) with promising results in patients (Edmonds et al. 1994). Ferro-Flores et al. also developed ^166^Dy/^166^Ho-labelled hydroxide macroaggregates (HM) for radiation synovectomy. For this, ^164^Dy_2_O_3_ was neutron activated which was then converted into ^166^DyCl_3_ and the ^166^Dy isotope purified. The ^166^DyCl_3_ was then incubated with a sodium hydroxide solution in an ultrasonic bath to form ^166^Dy/^166^Ho-labelled HM with sizes ranging from 2 to 5 μm and more than 99.5% of radiochemical purity. In vivo studies in rats showed that the ^166^Dy/^166^Ho-labelled HM were retained at the administration site even after 7 days (Ferro-Flores et al. 2004).

## Miscellaneous applications

### Intraluminal irradiation

Another application of ^166^Ho is for intraluminal irradiation, in which typically stenoses or other blood vessel wall abnormalities are treated. Different approaches for intraluminal irradiation have been studied, e.g. ^166^Ho –impregnated polyurethane coating on a stent (Won et al. 2002; Won et al. 2005), ^166^Ho-coated surface of a dilatation balloon (Kim et al. 2003; Hong et al. 2003) or dilatation balloons filled with ^166^Ho conjugated to DTPA (Hong et al. 2004; Park et al. 2007; Park et al. 2003; Kim et al. 2000). Won et al. developed self-expandable stents covered with ^166^Ho in a canine model. Both studies demonstrate the formation of fibrosis on the adjacent wall. No serious complications have been reported and it was indicated that this type of stent could be used as an alternative treatment (Won et al. 2002; Won et al. 2005). Hong et al. developed a procedure in which the surface of a dilatation balloon could be coated with ^166^Ho. Herein is was stated that coated balloons are more resistant to leaking radioactivity as opposed to liquid [^166^Ho]Ho DTPA filled balloons and a higher dose may be delivered. However, there may be a chance of peeling off of the ^166^Ho-coated surface and the activity can be unevenly distributed (Hong et al. 2003). Kim et al. delivered a dose of 20 Gy, using a dilatation balloon with ^166^Ho coated on the surface, in a porcine coronary stent restenosis model and the irradiated group demonstrated a significantly decreased stenosis area compared to the control group (Kim et al. 2003). As already mentioned, ^166^Ho is also used in combination with DTPA. In a study in rats and rabbits it was shown that after intravenous injection [^166^Ho]Ho DTPA was excreted relatively quickly to the urine bladder. Furthermore, a relatively low absorption in vital organs was seen (Hong et al. 2002). In a phantom study, in which clinically relevant irradiation and duration of exposure were tested, it was shown that [^166^Ho]Ho DTPA is a good source for endovascular irradiation (Joh et al. 2000). This was confirmed in a study in 12 pigs, in which an average dose of 30 Gy was delivered to treat pseudointimal hyperplasia (Park et al. 2007). The feasibility of the treatment to prevent restenosis was also demonstrated by Kim et al. in an animal study on 34 pigs (Kim et al. 2000). Park et al. conducted a study on 56 patients with in-stent restenosis. A balloon filled with liquid [^166^Ho]Ho DTPA which was a little longer than the stenosed stent was placed inside the lesion. For all patients the treatment was successful, and in 93% of the patients no restenosis occurred within the time of follow-up (approximately 19 months) (Park et al. 2003). Hong et al. tested whether it would be beneficial to combine [^166^Ho]Ho DTPA with a CT contrast medium. It was demonstrated that a CT contrast medium may have an added value to detect radiation leakage of the balloon on real-time basis (Hong et al. 2004). Majali et al. studied the potential of [^166^Ho]Ho dimethyl di ethylene triamine penta acetic acid (DMDTPA) for intraluminal irradiation in mice. Biodistribution data demonstrated a faster clearance of the complex compared to [^166^Ho]Ho DTPA, almost no accumulation in bone or any other vital organs was seen (Majali et al. 2002).

### Patches

**Fig. 5:**
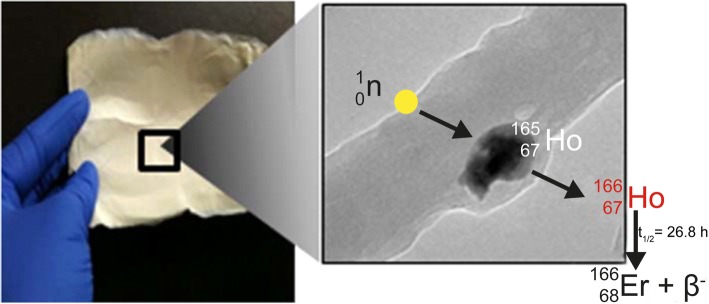
Holmium polyacrylonitrile patch. Reprinted (adapted) with permission from Munaweera et al.. Radiotherapeutic bandage based on electrospun polyacrylonitrile containing holmium-166-labelled iron garnet nanoparticles for the treatment of skin cancer, Copyright 2014 American Chemical Society

Patches for the treatment of skin cancer and Bowen’s disease (a precursor of squamous cell skin cancer) containing ^166^Ho have also been fabricated. Lee et al. created patches in which ^165^Ho-labelled macroaggregates were attached to an adhesive tape followed by coating with a polyethylene film, where after the patch was neutron-activated. These were then applied to mice with induced skin tumours (Lee et al. 1997). Chung et al. also created a patch to treat skin malignancies by dissolving holmium-nitrate and polyurethane in a solvent mixture of dimethylformamide and tetrahydrofuran and the solution was casted on an aluminium dish until solvent evaporation and consequent formation of a dried film. Particles of 30 to 50 μm were obtained from this cast, neutron activated and afterwards attached to adhesive tape. These patches were applied to patients with Bowen’s disease (Chung et al. 2000). The results of both studies were positive and complete remission was observed. However, they also described several disadvantages, such as the limited penetration depth of the beta radiation, which makes it hard to irradiate bulky tumours. Another disadvantage of patches to which macroaggregates are attached is the chance of isotope leakage, which may lead to contamination. Manuweera et al. developed a bandage of nanofibers containing homogeneously incorporated holmium nanoparticles (Munaweera et al. 2014). First, ^165^Ho-labelled iron garnet nanoparticles (^165^HoIG) were prepared by mixing iron nitrate and holmium nitrate with ethylene glycol followed by precipitation of the ^165^HoIG nanoparticles with sodium hydroxide. This resulted in nanoparticles with a length of 55 nm and a width of 28 nm. Then, electrospinning of a solution containing polyacrylonitrile (PAN) and the ^165^HoIG nanoparticles dispersed in dimethylformamide resulted in a bandage containing nanofibers with the nanoparticles incorporated (Fig. 5). The ^165^HoIG/PAN nanofibers were neutron activated and showed to be stable up to a thermal neutron-flux of approximately 3.5 × 10^12^ neutrons/cm^2^ s for at least 4 h at 100 °C. Stability studies using simulated body fluid revealed no leakage of the nanoparticles from the nanofibers after 8 h incubation (Munaweera et al. 2014).

### Radiovertebroplasty

In order to treat bone malignancies a new type of treatment named radiovertebroplasty has been developed. In this therapy, radionuclides are incorporated in bone cement to generate a local high dose. For example, Hirsch et al. developed an acrylic cement of polymethyl methacrylate (PMMA) and uniformly incorporated ^166^Ho. The authors concluded ^166^Ho is a potential radionuclide for radiovertebroplasty based on dosimetry (Hirsch et al. 2008). Donanzam et al. managed to generate stable bioceramics using the sol-gel technique (see section 3.2) in which ^166^Ho is incorporated in calcium phosphate. Despite the described potential of the developed biomaterial, clinical trials are necessary to further investigate the bioactivity and efficacy in bone malignancies (Donanzam et al. 2013).

## Conclusion

In this review, we have described the broad spectrum of medical applications of ^166^Ho as a result of the growing research interest over the years. ^166^Ho is among the most promising relatively new therapeutic radioisotopes. Holmium has very interesting physical properties: a high energy beta particle emission for treatment, a low energy gamma photon emission for imaging and a high magnetic susceptibility for MR imaging. Therefore, ^166^Ho labelled systems are attractive for radionuclide treatment. Furthermore, because of its natural abundancy of 100% and a high cross section of 64 b it can be produced in a straightforward manner, both fast and therefore relatively low-priced. In the radionuclide therapies, it is observed that quantitative analyses and dosimetry are gaining more and more importance to predict treatment efficacy. The value of treatment verification as an integral part of any radiotherapeutic treatment is also underpinned by the Council Directive 2013/59/EURATOM, which came into effect in February 2018, stating: *“For all medical exposure of patients for radiotherapeutic purposes, exposures of target volumes shall be individually planned, and their delivery appropriately verified …”*. Imaging and dosimetry can be used for better patient selection and to improve treatment dose calculations. The multimodal imaging medical isotope ^166^Ho can be a good response to the increasing demand of more personalized treatments in patients.

## Data Availability

N.a.

## Abbreviations

^166^Ho-SD: A scout dose of ^166^Ho-MS
^99m^Tc-MAA: Technetium-99 m-labelled macro-aggregated albumin
AcAc: Acetylacetonate
APDDMP: N,N- dimethylenephosphonate- 1-hydroxy-4-aminopropylidenediphosphonate
CT: Computed tomography
Dex: Dextran hydrogel
DMDTPA: Di ethylene triamine penta acetic acid
DMF: Dimethylformamide
DOTMP: 1,4,7,10-tetraazacyclododecane-1,4,7,10-tetramethylene-phosphonic acid
DTPA: Diethylenetriaminepentaacetic acid
EBRT: External beam radiotherapy
EDTMP: Ethylene-diamine-tetramethylene phosphonic acid
FHMA: Ferric hydroxide macroaggregates
HCC: Hepatocellular carcinoma
HM: Hydroxide macroaggregates
mAbs: monoclonal antibodies
MOF: Metallic organic framework
MRI: Magnetic resonance imaging
MS: Microspheres
PAM: Pamidronate
PAN: Polyacrylonitrile
PLLA: Poly(L-lactic acid)
PMMA: Polymethyl methacrylate
REILD: Radioembolization induced liver disease
SIRT: Selective internal radiation therapy
SPECT: Single photon emission computed tomography
TTHMP: Triethylene tetramine hexa (methylene phos- phonic acid)
